# A fatal respiratory complication of malaria caused by *Plasmodium vivax*

**DOI:** 10.1186/s12936-023-04720-1

**Published:** 2023-10-09

**Authors:** Angie R. López, Ezequias B. Martins, Anielle de Pina-Costa, Ana Beatriz Pacheco-Silva, Marcel T. Ferreira, Roxana F. Mamani, Paula J. T. Detepo, Otilia Lupi, Clarisse S. Bressan, Guilherme A. Calvet, Michele F. B. Silva, Maria de Fátima Ferreira-da-Cruz, Fernanda de Bruycker-Nogueira, Ana Maria B. Filippis, Cláudio Tadeu Daniel-Ribeiro, André Siqueira, Patrícia Brasil

**Affiliations:** 1 Instituto Nacional de Infectologia Evandro Chagas Fiocruz, Rio de Janeiro, Brazil; 2 Centro de Pesquisa, Diagnóstico e Treinamento em Malária da Fiocruz e da Secretaria de Vigilância em Saúde, Ministério da Saúde, Brasília, Brazil; 3 Laboratório de Pesquisa em Malária, Instituto Oswaldo Cruz, Fiocruz, Av. Brasil, 4365, Manguinhos, Rio de Janeiro, 21045-900 Brazil; 4 Laboratório de Flavivirus, Instituto Oswaldo Cruz, Fiocruz, Rio de Janeiro, Brazil

**Keywords:** Malaria, *Plasmodium vivax*, Respiratory, Fatal complication

## Abstract

**Background:**

Malaria is endemic and represents an important public health issue in Brazil. Knowledge of risk factors for disease progression represents an important step in preventing and controlling malaria-related complications. Reports of severe forms of *Plasmodium vivax* malaria are now becoming a common place, but respiratory complications are described in less than 3% of global literature on severe vivax malaria.

**Case presentation:**

A severe respiratory case of imported vivax malaria in a previously healthy 40-year-old woman has been reported. The patient died after the fifth day of treatment with chloroquine and primaquine due to acute respiratory distress syndrome.

**Conclusions:**

Respiratory symptoms started 48 h after the initiation of anti-malarial drugs, raising the hypothesis that the drugs may have been involved in the genesis of the complication. The concept that vivax malaria is a benign disease that can sometimes result in the development of serious complications must be disseminated. This report highlights, once more, the crucial importance of malaria early diagnosis, a true challenge in non-endemic areas, where health personnel are not familiar with the disease and do not consider its diagnosis promptly.

## Background

Malaria is an endemic disease in Brazil, and *Plasmodium vivax* is the most prevalent causing species, implicated in over 80% locally diagnosed cases. *Plasmodium vivax* infection is usually associated with benign presentations of malaria. However, serious manifestations with potentially kidney injury, acute respiratory distress syndrome (ARDS), splenic rupture, and even cerebral malaria have been reported. The underlying mechanisms of such severe manifestations are not well understood [1]. Therefore, vivax malaria is emerging as a potentially serious disease associated with a broad spectrum of complications and significant changes in laboratory parameters, indicating a recent increase in virulence [2]. It is well documented that *P. vivax* can cause severe forms of malaria that are potentially fatal [1–3].

## Case presentation

A healthy 40-year-old female Brazilian patient residing in the state of Rio de Janeiro reported a trip to São Gabriel da Cachoeira (Amazon State) between October 18, 2019 and November 26, 2019. On November 30, 2019, she presented to a general hospital in Rio de Janeiro with malaise, myalgia, and arthralgia. On December 2nd, 2019 she presented high fever, accompanied by chills and sweating. The patient sought medical care and was medicated with analgesics and antithermics for treatment support of a possible nonspecific viral infection. The case evolved with periods of recurrent fever.

On December 6, 2019 (6 days after the onset of symptoms), she was admitted to the Emergency Department with a severe headache, anaemia (haemoglobin: 7.8 g/dL), heavy vaginal bleeding, and high fever. Vaginal bleeding continued for 5 days and severe thrombocytopenia was reported observed (33,000/µL). The patient was not pregnant as she had previously undergone a uterus tubal ligation. She had no history of using contraceptives. She left the hospital on December 12, 2019, without bleeding and with improved thrombocytopenia (116,000/µL).

On December 13, 2019 (13 days after the onset of symptoms), the patient was admitted to the Infectious Diseases ward of the *Fundação Oswaldo Cruz*, with moderate fever, headache and malaise, where the diagnosis of vivax malaria primary infection was made (3720 parasites/mm^3^). Treatment with chloroquine for 3 days (600 mg—first day/450 mg—second and third days) and primaquine (30 mg/day—7 days) was started for the patient who weighed 79.4 kg. Vaginal bleeding stopped without recurrence and platelets increased to 144,000/µL. On the same day (December 13, 2019), the patient was discharged from the outpatient clinic. On discharge, she still had fever, but did not present any sign of severe illness. It was recommended that she return for a follow-up consultation for clinical and laboratory evaluation.

Three days after beginning treatment, the patient reported myalgia, high fever, severe headache, and progressive tachypnea (54 breaths per minute). A blood smear exam and reverse transcriptase polymerase chain reaction (RT-PCR), were performed, both of which were found to be negative for the presence of malaria parasites on December 16, 2019.

Physical examination (December 16, 2019) showed moderate hepatosplenomegaly, lungs with bilateral crackling rales, and severe anaemia. Blood gas tests revealed progressive hypoxia (oxygen saturation: 62%). A chest X-ray showed bilateral interstitial infiltrates. A diagnosis of severe ARDS was confirmed (low arterial oxygen pressure, increased arterial carbon dioxide pressure, and low oxygen saturation) the following day, and the patient was admitted to the Intensive Care Unit. Special care was initiated and intravenous artesunate (2.4 mg/kg) was prescribed because the possibility of recurrence was considered, despite negative parasitaemia. The patient developed progressive worsening of tachypnea and low blood oxygenation, requiring orotracheal intubation. Antibiotic therapy with ceftriaxone and azithromycin was started to treat possible nosocomial bacterial pneumonia. Whole blood collected prior to antibiotic therapy was culture negative. Intensive care was not enough to reverse the metabolic changes, however, and the patient died on December 17, 2019 following three episodes of cardiac arrest.

Diagnostic tests for HIV infection were negative. Serum samples from the 18th day after the onset of symptoms had negative RT-qPCR results for Dengue virus (DENV), Chikungunya virus (CHIKV), and Zika virus (ZIKV). Serological test performed for CHIKV IgM and IgG antibodies, were positive in the serum from the 18th day after the onset of symptoms. Non-reactive anti-DENV-IgM and IgG serological test were confirmed.

**Fig. 1:**
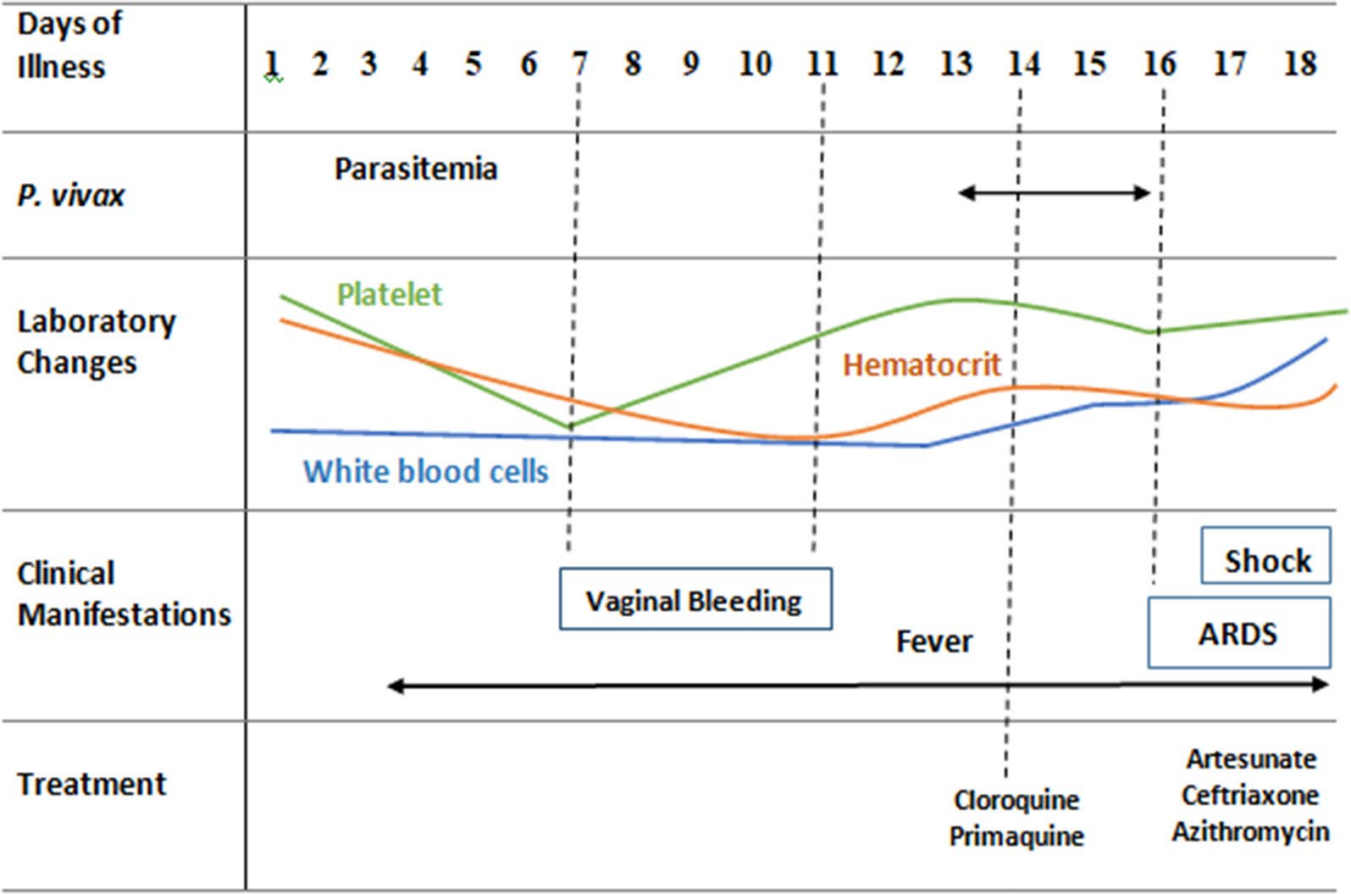
Main clinical phenomena leading up to death

A progressive increase in the patient’s leukocytes and C-Reactive Protein suggested worsening of the inflammatory and infectious injury. Laboratory tests performed during treatment showed severe anaemia, mild thrombocytopenia, and a moderate increase in blood creatinine. Liver function was normal throughout the treatment follow-up (Table 1). Figure 1 describes the main biological parameters registered during the 18 days of illness preceding the patient’s death.

**Table 1:** Laboratory tests performed during treatment

	Dec 13, 2019	Dec 15, 2019	Dec 16, 2019	Dec 17, 2019
Haemoglobin (g/dL)	9.2	7.4	7.0	6.8
Haematocrit (%)	26.1	20.8	19.4	20.4
White blood cells (×10^3^/µL)	5560	8140	8730	11,380
Platelets (×10^3^/µL)	144,000	124,000	266,000	127,000
Alanine aminotransferase (IU/L)	38	29	28	74
Alkaline phosphatase (IU/L)	51	33	28	73
Creatinine (mg/dL)	0.69	0.66	0.57	1.26
C-Reactive protein (mg/dL)	8.90	9.02	10.78	18.52
G6PD deficiency			Negative	
Anti-CHIKV-IgM				Detected
Malaria (blood smear)	*P. vivax* 3720 parasites/mm^3^	Negative		Negative

## Discussion and conclusions

It is troubling that the diagnosis of malaria was made a full 13 days after symptom onset, and 17 days after the traveller returned from the Amazon, which is the main area of transmission in Brazil that is responsible for 99% of malaria cases annually. Health professionals outside malaria endemic areas should be trained to investigate patients’ travel history, to optimize early diagnosis and adequate treatment of malaria and prevent morbidity and fatal outcomes [4].

According to a review by Val et al. [5], the prevalence of ARDS among vivax malaria cases ranged from 2.2% (adults) to 2.8% (children), with a mortality rate around 50%. Matlani et al. [2] described a cohort of 177 patients with vivax malaria in India where they found a high proportion of severe disease complications (32.7%), but the study subjects were predominantly children (56%) and no severe respiratory complications were observed. In a previous study in India conducted mostly on hospitalized adult patients, ARDS was described in 42.5% of cases of vivax malaria [6]. In a study conducted in Brazil on 587 vivax malaria hospitalized patients, respiratory complications were described in 5.1% (30 patients) of the cohort, 43.3% (13 patients) of whom progressed to severe disease and 16.6% (five patients) died, greatly exceeding a case fatality rate of just under 1% [3, 7]. The patient in our case had no previous lung disease, so respiratory complications were not expected. The clinical manifestations present at the time of malaria diagnosis were compatible with benign evolution, allowing for at-home treatment. The haemorrhagic phenomenon occurred in a short period and resolved in 5 days, after a progressive increase in platelets. The decrease in platelets was probably not due to low medullary production. Probably, platelets were trapped in some organs (such as the spleen) during the intense inflammatory process, which would explain the rapid increase in platelets, although an underlying viral illness can not be excluded.

As expected, the RT-PCR results for a panel of arboviruses made 18 days after the onset of symptoms were negative, since viraemia tends to be lower after the acute phase of the disease. On the same day serological tests performed for IgM and IgG antibodies to CHIKV were positive in the patient’s serum (low reactivity). Previous CHIKV infection could not be confirmed as the first and only serum sample did not allow the comparison of titers of immunoglobulin from a single sample, a well-known limitation of serological tests [8]. It is possible that the presence of antibodies against CHIKV resulted from a cross-reactive immune response to plasmodium and CHIKV. In a study conducted by Morch et al. [9], it was reported that positive serological tests for dengue, leptospirosis, scrub typhus, and chikungunya were common in patients with malaria and bacteraemia both confirmed by molecular methods, suggesting low specificities of the serological tests. These limitations of serological diagnostic tests should be taken into consideration when approaching a patient with acute undifferentiated fever in countries endemic to malaria and arboviruses, such as Brazil. Furthermore, the polyclonal lymphocyte activation seen in malaria, has already been implicated in the genesis of positive serological tests not related to malaria or coinfections and should also be considered [10].

The pathogenic mechanisms associated with the respiratory complications of vivax malaria are still unclear. In a study that followed 30 patients with respiratory complications, respiratory symptoms were observed after the initiation of anti-malarial drugs [3]. Val et al. [5] performed an important review, reporting the development of respiratory complications after starting treatment. Anstey et al. [11] hypothesized that anti-malarial drugs can be followed by an inflammatory process in the pulmonary microvasculature, increasing capillary permeability and leading to respiratory failure. In this same study, they found a decreased vascular component of lung gas transfer which might be explained by vivax parasite sequestration. Post-treatment, a progressive decline in the alveolar-capillary component of gas transfer was greater in the vivax vs. falciparum patients, consistent with a greater post treatment inflammatory response in the vivax patients [11].

In a study with 79 patients diagnosed with falciparum malaria with respiratory complications, 42% had the complication within the first 48 h after starting treatment and 46% after 48 h, when parasitaemia was falling [12]. Our patient did not report respiratory symptoms before starting anti-malarial drugs. After the third day of treatment with chloroquine and primaquine, the diagnosis of ARDS was confirmed, progressing to death after the fifth day of treatment initiation. The introduction of anti-malarial drugs may have intensified the development of a potent inflammatory reaction in lung tissue after the acute phase of malaria. The use of anti-malarials may have triggered respiratory complications, as suggested by Anstey, who conjectured that a decrease in the vascular component of lung gas transfer could be explained by vivax parasite sequestration [11].

Some studies point to the ability of *P. vivax* to cause cytoadherence of red blood cells, which could explain the potential to develop severe manifestations, but this characteristic is by far more observed in *Plasmodium falciparum* infections [13, 14]. The cytoadherence of red blood cells could be a hypothesis for the sudden development of respiratory failure, but necropsy was not authorized by the patient’s family.

For endemic malarial regions, some risk factors may be implicated as disease severity criteria, such as patient age (age extremes), pregnancy, presence of comorbidities, and disease relapses [15]. The patient was middle aged, had no comorbidities, and was not pregnant. This was the patient’s first *P. vivax* infection. The patient in the case report did not have any risk factors associated with progression to severe disease. However, the delay in diagnosis and in starting specific treatment might have contributed to the poor outcome.

Respiratory complications should be considered in vivax malaria, especially after delayed diagnosis and treatment. Further studies are needed to elucidate the mechanisms underlying such severe complications, to avoid fatal outcomes. Daily monitoring during treatment is mandatory for early identification of complications to facilitate rapid clinical support. Travel history to malaria-endemic areas should be rigorously investigated, with the aim of early diagnosis and adequate treatment of malaria.

## Data Availability

Not applicable.

## Abbreviations

ARDS: Acute respiratory distress syndrome
RT-PCR: Reverse transcriptase polymerase chain reaction
DENV: Dengue virus
CHIKV: Chikungunya virus
ZIKV: Zika virus
